# Novel Primary Immunodeficiency Candidate Genes Predicted by the Human Gene Connectome

**DOI:** 10.3389/fimmu.2015.00142

**Published:** 2015-04-01

**Authors:** Yuval Itan, Jean-Laurent Casanova

**Affiliations:** ^1^Rockefeller Branch, St. Giles Laboratory of Human Genetics of Infectious Diseases, The Rockefeller University, New York, NY, USA; ^2^Necker Branch, Laboratory of Human Genetics of Infectious Diseases, INSERM U1163, Paris, France; ^3^Imagine Institute, University Paris Descartes, Paris, France; ^4^Howard Hughes Medical Institute, New York, NY, USA; ^5^Pediatric Hematology-Immunology Unit, Necker Hospital for Sick Children, Paris, France

**Keywords:** candidate gene identification, primary immunodeficiencies, the human gene connectome, disease genomics, computational biology

## Abstract

Germline genetic mutations underlie various primary immunodeficiency (PID) diseases. Patients with rare PID diseases (like most non-PID patients and healthy individuals) carry, on average, 20,000 rare and common coding variants detected by high-throughput sequencing. It is thus a major challenge to select only a few candidate disease-causing variants for experimental testing. One of the tools commonly used in the pipeline for estimating a potential PID-candidate gene is to test whether the specific gene is included in the list of genes that were already experimentally validated as PID-causing in previous studies. However, this approach is limited because it cannot detect the PID-causing mutation(s) in the many PID patients carrying causal mutations of as yet unidentified PID-causing genes. In this study, we expanded *in silico* the list of potential PID-causing candidate genes from 229 to 3,110. We first identified the top 1% of human genes predicted by the human genes connectome to be biologically close to the 229 known PID genes. We then further narrowed down the list of genes by retaining only the most biologically relevant genes, with functionally enriched gene ontology biological categories similar to those for the known PID genes. We validated this prediction by showing that 17 of the 21 novel PID genes published since the last IUIS classification fall into this group of 3,110 genes (*p* < 10^−7^). The resulting new extended list of 3,110 predicted PID genes should be useful for the discovery of novel PID genes in patients.

## Introduction

Germline mutations are being found to underlie an increasing number of primary immunodeficiency (PID) diseases. With current advances, and the improved quality and decreasing cost of high-throughput sequencing (HTS), it is now possible to detect the full set of gene variants in PID patients, through techniques such as whole-exome sequencing (WES) or whole-genome sequencing (WGS). The genome of each patient contains about 20,000 coding variants and hundreds of thousands of non-coding variants (1–3). It is not straightforward to identify the PID-causing gene from the HTS data for a patient (4, 5), and the most widely used approach involves selecting known disease-causing genes as candidate genes for further investigation. Thus, in this approach, variants are identified as candidate PID-causing genes only if they concern genes already listed among those known to cause PIDs (229 such genes have been identified to date) (6, 7). This approach is simple to implement, but is likely to miss the true PID-causing gene in many PID patients, as novel PID genes (i.e., not already included among the 229 genes known to cause PIDs) would potentially be ignored. Alternatively, the investigator would face the non-trivial task of inferring relevant PID-candidate genes from a list of hundreds following rigorous variant-level filtering, mostly by estimating potential biological functional relatedness to the known PID genes.

To tackle the above problem, we recently described the human gene connectome (HGC)^1^ as the set of biological distances and routes (i.e., genes located between two genes) between all human genes, predicted *in silico* by a shortest distance algorithm applied to the full human genome network, conceptually similar to GPS navigation (5). The HGC and its associated user-friendly server (8) could be used to detect new candidate PID-causing genes, by ranking all genes harboring variants in PID patients on the basis of their biological proximity to already known PID genes, assuming the most highly ranked genes to be the most likely to cause PIDs.

However, a list of novel potential PID-causing gene candidates, rigorously identified on the basis of biological relevance to the PID phenotype (both by HGC-predicted biological distance to PID-causing genes and by the relevance of the candidate gene’s biological function to PID), would be useful and easy for most PID investigators to use. As the HGC-predicted gene candidates may include some false positives, due to the prediction algorithm being based on protein–protein binding interactions rather than biological functions associated with the gene, it is important to filter gene lists further, according to biological relevance, at the final stage of *in silico* prediction.

We generated a list of *in silico*-predicted novel PID-causing gene candidates, which we describe here. We first determined the biological clustering level of all PID genes, then used the HGC to extract the top 1% of all human genes biologically closest to all known PID genes. Finally, we selected as PID gene candidates only those genes with relevant biological functions, similar to those of known PID genes. We also show that the list of novel PID-causing gene candidates generated contains 17 of 21 recently reported PID-causing genes (not included in the database used for prediction purposes in this study).

## Materials and Methods

### Biological proximity of PID genes: Statistical simulations

We determined whether the 229 known PID-causing genes (6) were significantly closer to each other, biologically, than to other genes, by estimating the biological distances between all these genes (a total of 22,366 biological distance values) with the HGC and associated server (5, 8)^2^. We then randomly sampled one million biological distances from the set of all human genes (9), and determined, for both the PID and all-gene sets, the proportion of distances falling into the following categories: (1) small biological distances (<10); (2) small-medium biological distances (between 10 and 20, 20 being the median biological distance between all human genes); (3) medium-large biological distances (between 20 and 30); (4) large-very large biological distances (between 30 and 40); and (5) very large-extremely large biological distances (>40). We then compared the biological density of PID genes with that of other human genes, by first calculating the median distance between all 229 known PID genes, and then simulating sets of 229 randomly chosen genes, calculating the median distance between these genes, and estimating a *p*-value by determining the proportion of simulated random gene sets with a median biological distance smaller than that for the known PID genes.

### Initial extraction of novel PID-candidate genes

We used, as an input, the connectomes (see text footnote 1) of the 229 known PID-causing genes (6), a gene-specific connectome being defined as the set of all human genes ranked according to their biological proximity to the gene of interest. From these 229 gene-specific connectomes, we extracted only the genes in the top 1% of all human genes by *p*-value, to obtain an initial list of 29,885 genes. We then processed the novel candidate gene data in two steps, to prevent redundancy: (1) we removed all the predicted genes already in the list of 229 known PID-causing genes; and (2) we retained only one instance per novel PID-candidate gene. These two filtering steps decreased the list to 5,012 non-redundant genes.

### Filtering of PID gene candidates by functional enrichment

We then narrowed the list of genes down to those of biological relevance, by hypothesizing that a novel PID-causing gene would be likely to have a biological function similar to those of already known PID genes. For example, the gene *CPN2* was predicted by the HGC biological distance to PID core gene *IKBKG* to be a PID candidate. However, *CPN2* has the biological function of protein stabilization, which is not a biological function that is enriched in known PID genes; therefore, *CPN2* could plausibly be removed from the initial list of PID-candidate genes. We therefore first used DAVID (10) to estimate the gene ontology (GO) biological functional enrichment of all known 229 PID genes. We selected only those functions for which *p* < 0.05 (a total of 462 GO terms). We then applied DAVID GO biological terms analysis (11) to the PID gene candidates, and selected only genes associated with at least 1 of the 462 PID GO terms. This resulted in a final list of 3,110 *in silico*-predicted novel candidate PID genes.

### Phylogeny of known and estimated novel PID genes

The biological-interrelatedness between the 229 known PID genes and the 3,110 candidate PID genes predicted in this study was estimated with the functional genomics alignment (FGA) phylogeny (5). We first created a biological distance matrix between all known and predicted PID genes; we then applied a neighbor-joining algorithm, in the R statistical programing language APE (12) (analyses of phylogenetics and evolution) package nj function. Finally, we plotted a phylogenetic fan-shaped tree based on HGC-predicted biological distances between known and predicted PID genes, using the plot function of the APE package (12).

### Generation and plotting of gene networks

For visual depiction of the distribution of the 229 currently known PID genes within the full human genome, we used the predicted HGC biological distance between all human genes, selecting only direct biological connections (5, 13) between genes, to lower the complexity of the network. We then used the NetworkX python package for complex network analyses and visualizations (14), applying the spring layout function, which estimates the localization of each gene in a two-dimensional space with the Fruchterman–Reingold force-directed algorithm (15). The nodes of known PID genes were plotted three times larger than those of the other human genes.

### Computing resources and programing languages

This project was performed on a Mac Pro machine with 12 cores and 128 GB RAM. Biological distances between genes were calculated by the HGC and server. Data extraction, statistical simulations, and network analyses and visualizations were performed with the Python programing language. FGA phylogenetic analyses and visualizations were performed with the R programing language for statistical computing^3^. The programs and online server used in this study (in particular for ranking candidate genes by biological distance from core genes and FGA trees generation) are freely available to non-commercial users with step-by-step instruction at http://lab.rockefeller.edu/casanova/HGC and http://hgc.rockefeller.edu, and the scripts for the minor technical procedures in this study are all available from the authors upon request.

## Results

### Distribution of PID genes within the whole human genome

**Figure 1:**
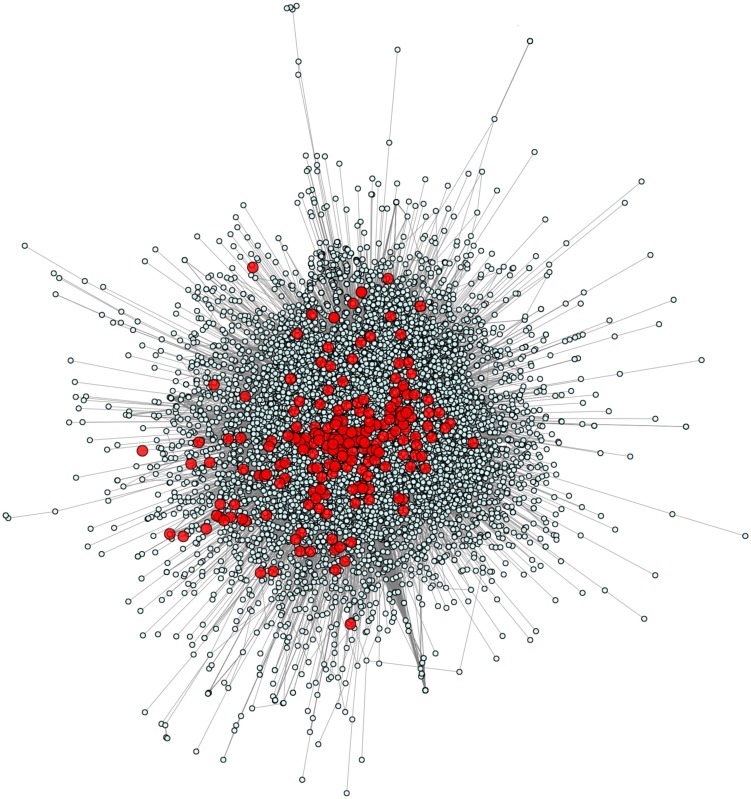
**The human genome and PID gene network**. This figure shows all 229 known PID-causing genes (red) together with all 14,131 human protein-coding genes for which HGC-predicted biological distance information was available.

We calculated and plotted the network of all 229 known PID-causing genes related to 50 PID syndromes (6) in the context of the full human genome (Figure 1). Using the HGC-predicted direct biological distance between human genes, we found that PID genes tend to be in the central hub of the human genome network, with only a small minority in the central hub’s periphery and none in the extreme periphery of the whole human genome. PID genes are forming several tightly intra-related sub-clusters (i.e., in most cases, a PID gene will have as a close functional neighbor at least one other PID gene) across a diversity of biological mechanisms, demonstrating the variety of genetic pathways underlying PIDs.

### Small biological distances between PID genes

**Figure 2:**
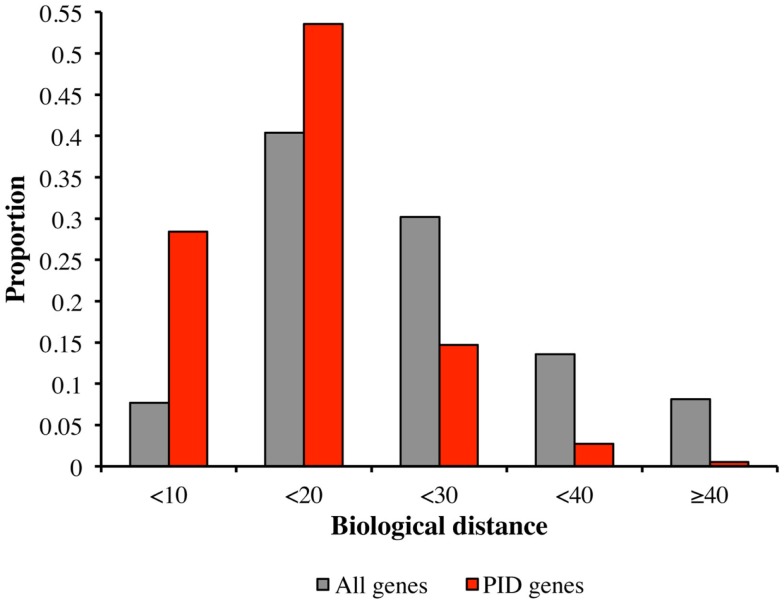
**Comparison of biological distances between PID genes and all human genes**. Biological distances between PID genes (red) and all human genes (gray), according to the proportion of distances in five categories: (1) small biological distance (<10); (2) small-medium biological distance (between 10 and 20); (3) medium-large biological distance (between 20 and 30); (4) large-very large biological distance (between 30 and 40); and (5) extremely large biological distance (>40).

We tested the hypothesis that PID genes are functionally close to each other (which would be the prerequisite for identifying candidate PID genes on the basis of biological proximity), by comparing the biological distances between PID genes and all human genes, in terms of the proportions of biological distances assigned to five categories, from the smallest to the largest (Figure 2). Most intra-PID gene distances belonged to the very small-to-medium categories (28.4 and 53.6%, respectively), the proportion of PID genes falling into these categories being larger than for all human genes (7.7 and 40.0%, respectively).

We found that the median biological distance between known PID genes was 12.1, whereas that between simulated sets of 229 random genes was 20.4. None of the simulated sets of random genes had a median smaller than that of known PID genes (*p* < 10^−7^), consistent with the hypothesis of tight functional interrelatedness between PID genes. While it was expected that genes belonging to the same pathway would display small biological distances between each other, confirming this hypothesis makes it possible to infer novel PID genes based on HGC-predicted biological distance to core PID genes.

### Initial assessment of novel PID genes

Based on the demonstration that PID genes display close biological proximity, we hypothesized that currently unidentified PID genes would be located at a small biological distance from known PID genes. We therefore acquired the gene-specific connectomes of all 229 known PID-associated genes and extracted only those for which *p* < 0.01 for connection to the respective PID gene (29,885 gene occurrences, many genes occur more than once due to their close biological distance to more than one known PID gene). We then removed redundant genes, and genes from the set of 229 known PID genes. This left us with 5,012 non-redundant genes not previously identified as PID genes.

### Final set of proposed novel PID-candidate genes

**Figure 3:**
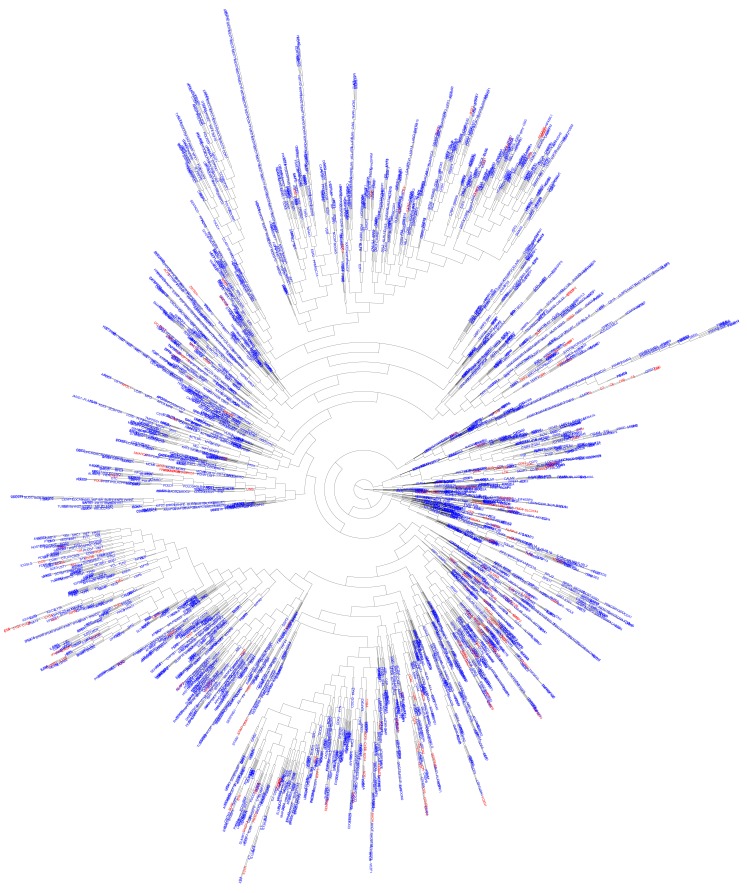
**Functional genomic alignment phylogeny of known PID genes and novel PID-candidate genes**. A phylogenetic tree of biological distances generated by the functional genomic alignment method, showing hierarchical clustering of all known (red) and predicted (blue) PID genes.

We hypothesized that most of the novel PID genes would be likely to have biological functions similar to those of known PID genes. We therefore used DAVID (10) to assess the functional enrichment, by biological GO, of all known PID genes. We retained only GO terms (11) for which *p* < 0.01 (Table S1 in Supplementary Material). We then applied biological GO terms analysis to the 5,012 candidate genes identified as described above, and extracted only those with a biological function already identified among known PID genes. This generated a final list of 3,110 *in silico*-predicted novel candidate PID genes, identified on the basis of biological distance from known PID genes and a similar biological function, and described in terms of their relatedness to their biologically closest PID gene (Table S2 in Supplementary Material). We then carried out hierarchical clustering of all known and predicted PID genes (5, 12). This analysis showed that the candidate PID genes identified in this study were evenly distributed over the whole range of known PID genes (Figure 3), while they form together a network that is tightly itra-related biologically and functionally (median biological distance of 11.0, compared to 20.4 between two random human genes, *p* < 10^−7^).

### Assessing the predictive power of novel PID-candidate genes

We retrospectively demonstrated the utility of this approach for identifying novel candidate PID genes, with 21 PID genes that were recently shown experimentally to cause PIDs (7, 16–38). None were included in the list of 229 known PID genes used in this study. Seventeen of these 21 genes were included in our list of proposed 3,110 novel PID-candidate genes, with *p* < 10^−7^ (estimated by random sampling computer simulations). Moreover, similar prediction rate of 17 of 21 is achieved with the extended list of 5,012 candidates, demonstrating the usefulness of the PID gene candidates short-listing procedure presented in this study. Table 1 describes these 17 genes in terms of their relatedness to the PID gene from the connectome of which they were identified.

**Table 1:** **New PID genes predicted from the list of novel PID-candidate genes**.

Predicted PID gene candidate	Known PID gene candidate	Biological distance between candidate and known	Rank of candidate in known	*p*-Value (percentile) of candidate in known	Route between candidate and known
BCL10	CARD11	1.001	1	0.00007	CARD11 ↔ BCL10
IRF7	MYD88	1.001	1	0.00007	MYD88 ↔ IRF7
IL21	IL21R	1.001	1	0.00007	IL21R ↔ IL21
CTLA4	ICOS	1.616	3	0.00021	ICOS ↔ CTLA4
STING (TMEM173)	TBK1	1.001	3	0.00021	TBK1 ↔ TMEM173
NFKB1	NFKBIA	1.001	4	0.00028	NFKBIA ↔ NFKB1
NLRC4	NOD2	1.183	5	0.00035	NOD2 ↔ NLRC4
NIK (MAP3K14)	CD40	1.064	8	0.00057	CD40 ↔ MAP3K1
TPP1	TINF2	1.191	18	0.00127	TINF2 ↔ TPP1
JAGN1 (JAG1)	CHD7	2.387	27	0.00191	CHD7 ↔ JAG1
TGFBR2	C8B	6.441	27	0.00191	C8B ↔ CLU ↔ TGFBR2
TGFBR1	C8B	6.441	28	0.00198	C8B ↔ CLU ↔ TGFBR1
INO80	ACTB	1.582	35	0.00248	ACTB ↔ INO80
DOCK2	RAC2	1.610	35	0.00248	RAC2 ↔ DOCK2
STAT4	STAT3	1.104	35	0.00248	STAT3 ↔ STAT4
ADA2 (TADA2A)	TBX1	7.228	83	0.00587	TBX1 ↔ C11orf30 ↔ TADA2A
IFIH1	FADD	4.141	87	0.00616	FADD ↔ MAVS ↔ IFIH1

*Showing the 17 (of 21) PID genes recently reported and identified in the list of PID-candidate genes described in this study*.

## Discussion

We describe here an extended list of 3,110 novel PID-candidate genes, initially predicted *in silico* with the HGC biological distance concept, then on the basis of the relevance of their biological function. We showed that PID genes (6) were significantly closer to each other biologically than other human genes. We then extracted the 1% of genes biologically closest to the known PID genes and further retained only those genes with a biological function similar to those known to display enrichment among PID genes. We generated a final *in silico*-predicted set of 3,110 human genes, which may be considered reliable candidate PID genes. In other words, we predict that there will be a high proportion of PID-causing genes among these 3,110 genes. We then demonstrated that 17 of 21 newly discovered PID genes were present in our proposed list of PID-candidate genes. We plan to use this list, together with the list of 229 known PID genes, in investigations of the HTS data for PID patients. A hit for one of these genes in this analysis would be associated with a higher likelihood of the gene being PID-causing.

It is important to note that as in any high-throughput genome-wide analysis, the choice of input data, algorithm, and even small fine-tuning is likely to strongly affect the outcome. However, we attempted here to provide a reliable prediction where false negatives (i.e., true PID genes that are not in the final provided list of candidate genes) are minimized – an aim that is expected to be achieved by following a biologically plausible hypothesis of the tight intra-relatedness of PID genes, together with having a small number of false positives, achieved by estimating the biological relevance of the gene candidates. The main limitation of the approach described in this study is that it is still expected to contain a large number of false positives: although the final list of 3,110 genes removes about 85% of irrelevant protein-coding genes, the function of a large proportion of human genes is poorly understood. Future advances in characterizing the functions of human genes and updates to genome-wide curated databases of gene ontologies and biological/genetic pathways should significantly improve the predictive power of our (and other *in silico*) described methodology.

The procedures described here could be used to infer novel disease gene candidates for other disease groups. For example, as information about known cancer driver genes is available (39)^4^, an extended list of cancer gene candidates could potentially be identified by HGC-predicted biological distance analysis of known cancer genes, to generate a final list of predicted novel candidates on the basis of biological function relevance. We stress that the predicted gene candidates (for PID and other diseases) should not be used for the purpose of excluding irrelevant genes, but rather to help investigators to identify novel disease-causing candidate genes. Moreover, as in any other *in silico* prediction methodology, in order for a mutation in a candidate gene to be confirmed as disease-causing, it must be verified by experimental immunological and genetic approaches, due to the complex nature of genetic disease pathogenesis, which in many cases involves phenotypic heterogeneity and incomplete penetrance, which current *in silico* methods cannot predict. We believe that rigorous use of the extended *in silico*-predicted list of candidate PID genes would increase the rate of novel PID-gene discovery in high throughput sequencing studies (40).

## Author Contributions

YI initiated the study, analyzed the data, and wrote the article. JC supervised the study, assisted with data resources, and wrote the article.

## Conflict of Interest Statement

The authors declare that the research was conducted in the absence of any commercial or financial relationships that could be construed as a potential conflict of interest.

## Supplementary Material

The Supplementary Material for this article can be found online at http://journal.frontiersin.org/article/10.3389/fimmu.2015.00142

Table S1 **Functional enrichment of known PID genes**. Biological GO enrichment categories (*p* < 0.05) for the 229 known PID genes. Other categories include GO accession term and number, fold-enrichment for the PID gene set for the specific GO term, and set of known PID genes for the specific GO term.

Table S2 **Proposed novel candidate PID genes**. The end result of this study is shown in 3,110 novel candidate PID-causing genes predicted *in silico* (column A). Column B corresponds to the known PID gene closest to the predicted novel PID gene. Other categories, by column: (C) biological distance between the known and predicted PID genes; (D) the rank of the predicted PID gene in the connectome of the known PID gene; (E) *p-*value for biological proximity of the predicted PID gene in the connectome of the known PID gene; (F) the predicted route (i.e., the genes on the shortest predicted route) between the known and predicted PID genes; and (G) the degrees of separation (i.e., the number of direct connections on the route) between the known and predicted PID genes.

## Footnotes

^1^http://lab.rockefeller.edu/casanova/HGC

^2^http://hgc.rockefeller.edu/

^3^http://www.r-project.org

^4^http://cancer.sanger.ac.uk/cancergenome/projects/cosmic/
